# Understanding the Functional Mobility of Adults with Developmental Coordination Disorder (DCD) Through the International Classification of Functioning (ICF)

**DOI:** 10.1007/s40474-018-0128-3

**Published:** 2018-02-06

**Authors:** Sally Scott-Roberts, Catherine Purcell

**Affiliations:** 1 Occupational Therapy, School of Health Care Sciences, College of Biomedical and Life Sciences, Cardiff University, Ty Dewi Sant, Heath Park, Cardiff, CF14 4XN UK; 2 School of Psychology, Early Years, Education and Therapeutic Studies, University of South Wales, Treforest, Pontypridd, CF37 1DL UK

**Keywords:** Developmental coordination disorder, Adults, Functional mobility, Falls, Phenomenological, International Classification of Functioning

## Abstract

**Purpose of Review:**

This phenomenological study explored the lived experience of six adults with developmental coordination disorder (DCD) and its potential impact on functional mobility. Utilising the International Classification of Functioning (World Health Organisation, 2001), the data derived from interviews were analysed to consider how persistent motor impairments impact on activity engagement and participation.

**Recent Findings:**

Much of the research evidence pertaining to DCD focuses on children. However, there is increasing acknowledgment that for some, the motor impairments synonymous with DCD continue into adulthood.

**Summary:**

The findings from this study suggest that for this group of participants, functional mobility can be compromised, restricting activity and participation. At a body structure/function level, participants identified additional impairments that moved beyond mobility, suggesting that the secondary consequences of fatigue and anxiety were disabling. However, personal factors were seen to mitigate some difficulties encountered to allow participants to remain actively engaged in a range of adult roles.

## Introduction

Developmental coordination disorder (DCD) is classified as a motor disability in the *Diagnostic and Statistical Manual of Mental Disorders*, Fifth Edition (DSM**-**5) [1]. Much of the research concerning the impact of living with this motor disability has focused on children; however, there is now a general recognition that DCD is a lifelong condition, with three quarters of children going on to experience difficulties in adulthood [2, 3]. Individuals who are diagnosed with DCD have difficulties with the learning of and the execution of effective and efficient motor skills, which can significantly impact upon a range of activities of daily living [4]. These difficulties exist in the absence of an underlying physical or neurological condition, intellectual delay or visual impairment [1]. Difficulties with both gross and/or fine motor skills described in childhood are reported to continue into adulthood and can have a negative impact on academic and vocational performance [5]. Clinical feedback from adults living with DCD suggests that they continue to have difficulties with balance and safe functional mobility. In a pilot study conducted by the authors, that compared age and gender matched adults with and without DCD, those with DCD were reported to trip and fall more frequently than their typical peers, which is in line with early research in the field [6]. The pilot study identified that 43% of adults with DCD had fallen more than 10 times in the previous 6 months with 80% tripping 1–5 times per week on average. What the survey did not fully explore was the impact of these trips and falls on the participants’ engagement and participation in a range of activities and life roles.

Difficulties with balance and as a consequence functional mobility^1^ have been identified as a key characteristic of this neurodevelopmental disorder [7]. Previous research that has analysed the walking patterns and static and dynamic balance control of children with a diagnosis of DCD indicates that compared to their typically developing peers, they present with gait instability [8•, 9]. Furthermore, poor postural control has been postulated as an explanation for the differences between the walking patterns of children with and without DCD [9]. This has, in part, been explained by deficits in neuromuscular activity such as problems with muscle strength, power and the force and timing of muscle contractions [10]. Whilst there is less research, there is an indication that adults who meet the diagnostic criteria for DCD continue to have difficulties with proficiency of movement compared to age-matched controls well into adulthood [5]. There is also a suggestion that adults with DCD are slower negotiating obstacles when walking compared to a typical control group, with a recent study showing a DCD group as having greater variability of movement whilst walking, when compared to a matched peer group [11•], suggesting that the proficiency of these groups functional mobility could be decreased.

The impact of living with DCD as an adult can go unnoticed, and as such, this neurodevelopmental disorder is often referred to as a ‘hidden disability’ [12]. The hidden nature of the characteristics of the disorder can preclude others from understanding how living with significant motor difficulties affects an individual’s ability to take an active role in a range of activities at home and in the wider community. To help explore the impact of living with such a motor disability, the International Classification of Functioning (ICF) [13] offers a framework that can be used to capture and classify information provided about an individual and their functioning. The framework has been designed to serve a variety of purposes, including providing a common language and conceptual understanding of the definition of health and disability. It also offers a systematic coding scheme to gather information about a population [13]. In the current study, it was used to frame our understanding of the experiences of adults with DCD. Functioning is defined in the ICF as the positive or neutral outcome of the interplay between an individual’s health condition and contextual concerns, such as the environment or personal factors [13]. It is an umbrella term used for body function/structure, activity and participation. When the interplay between the person’s health condition and the context produces a negative outcome, the umbrella term used is Disability. This is the overarching term that encompasses impairments, activity limitations and participation restrictions. As the purpose of this study was to explore the negative or disabling outcomes of living with significant motor difficulties that effect functional mobility, the primary aim was to explore how adult participants with a diagnosis of DCD view their impairments, activity limitations and participation restrictions.

## Method

As there is little known about the impact of enduring motor impairments on functional mobility, and as a consequence, activity and participation levels of adults with DCD, a phenomenological approach was adopted. It was considered that the utilisation of qualitative methods would gather rich information to ensure a deeper understanding of the phenomenon [14] from the individual’s perspective. In this case, it was used to explore the consequential effects of living with difficulties at a body function level on an individual’s ability to carry out activities of daily living and to participate in a range of life situations. The study received ethical approval from the University of South Wales (USW).

### Participants

Participants were recruited from a database of adult clients who had attended and been assessed for DCD by the multi-disciplinary team at the Dyscovery Centre, USW, within 5 years of the start of the project. During their clinical visit, all volunteer participants had provided signed agreement to indicate that they were happy to be approached for research studies being undertaken by the Dyscovery research team. This clinical recruitment strategy ensured that adult participants who took part had received a full multi-disciplinary assessment that explored all the criterion of the diagnostic criteria for DCD [1] and a diagnosis of DCD had been confirmed. The diagnostic assessment procedures had excluded participants who were found to have a degenerative or neuromuscular medical condition. In addition, participants were excluded from the study if, on screening, they were found to be on medication that could impact on their balance and/or motor skills, and/or had been identified as having difficulties with attention associated with a comorbid diagnosis of attention deficit hyperactivity disorder (ADHD).

Eligible participants were invited to take part in an in-depth interview and where applicable a second follow-up telephone conversation. A sample group of between 8 and 10 participants is often quoted as typical in phenomenological research [15]. In total, six participants took part in this study. This number was lower than initially intended but following the six interviews, the research team believed that theoretical saturation had been reached and no new themes were emerging from the interviews.

The six participants who took part in the study were all aged between 27 and 48 years of age and were all in employment, except one who was in full-time higher education. The sample comprised of five females and one male, all of whom met the inclusion criteria. Following the provision of detailed information about the study, all provided written informed consent to take part.

### Data Collection

The first author conducted one initial in-depth semi-structured interview with each of the participants, to explore their experiences of living with DCD and managing safe mobility. A pilot interview was conducted with one of the participants to ensure the questions elicited the depth of information required. Following this pilot interview, the term ‘functional mobility’ was defined prior to the interviews, no other changes were made.

The initial semi-structured interview encouraged participants to reflect on a typical day and specifically reflect on their experiences of navigating the various activities, roles and routines they regularly undertake, identifying any mobility challenges they may experience. The interviews ranged between 55 and 78 min in length. Following transcription of each interview, each participant was invited to review and make changes to the transcript; five of the six participants confirmed that the transcript was an accurate reflection of the interview and one made minor changes to the transcript. This member-checking process increased the trustworthiness of the data and also allowed the participants to expand on their reflections if they wished to do so. Where applicable, participants were also invited to take part in a follow-up individual telephone interview that focused on specific points, which had emerged from the first interview and required further explanation or exploration.

The semi-structured interview was audio recorded and transcribed verbatim and detailed field notes were taken during the telephone follow-up interview including the written recording of verbatim quotes. Participants, prior to becoming involved in the study, had understood and had given consent to anonymised verbatim quotes being used to authenticate their experiences in any future publication of findings.

### Data Analysis

The ICF [13], a framework for health and disability, was used to help analyse the information gathered during the semi-structured interviews. Utilising this framework to theme, the data can be considered a form of directed content analysis. Both researchers were involved in the initial analysis stage with both separately coding the scripts. Both brought different perspectives because of their professional backgrounds to the coding process, which did mean that some recoding was necessary so that both parties confirmed they were happy with the final analysis of the scripts.

Utilising the ICF framework allowed the researcher to identify from the participant own reports how their DCD and environmental factors interact and impact on daily life. In particular, it ensured a focus on the impact of participants’ motor impairments on safe functional mobility and as a consequence daily activities and participation in wider social situations.

## Findings

The umbrella term Disability refers to the negative outcome of a health condition such as DCD and its interplay with the environment (ICF) [13]. Through this framework, Disability can be viewed at three levels: impairments at a body function/structure level, activity limitation and participation restriction. To explore the impact of DCD, interview transcripts were reviewed and coded in light of the negative impact that motor difficulties synonymous with the disorder have at each of these three levels. As such, the findings will be presented in line with the ICF with pertinent verbatim quotes used to authenticate the summary of results.

### Impairments: Dysfunction at a Body Level

All of the participants discussed how they are regularly very aware of their difficulties with mobility and balance as they pursued daily activities. They often made the link between these impairments and their perceived increased chance of tripping and falling,

I am cautious now when out and about as I have so frequently tripped or missed the curb and twisted my ankle that I now have a weakness there.

All six participants spoke of becoming anxious about losing their balance and could explain how they often needed to exert conscious effort to stay on their feet.

I know if I loose concentration then I am likely to trip and fall when managing unfamiliar terrain.

Each participant had tried to make sense of their situation and had begun to analyse and identify their motor impairments, proposing that they had impairments in such things as body awareness and spatial perception.

I struggle with depth perception and this impacts on things like stairs.

I am not good at making judgements about where my body is in space and I think that increases my chances of bumping into others and things.

Many discussed how they had tried to address these impairments, for example:

I have poor core stability but have been trying to work on this in the gym to try and help my balance.

Interestingly, some on further analysis had been able to identify that increased anxiety pertaining to mobility was having a negative impact on their safety when walking.

My gait pattern shortens when I am anxious walking on uneven ground – but this I think probably puts me more of risk of falling.

In addition to the impact on mobility of the more obvious motor impairments associated with DCD, the participants spoke at length about how their energy levels were depleted by the effort having to be afforded to safely navigate their way around the environment.

My energy levels are sapped – sometimes think I must be lazy because others seem to fit more into their day.

Fatigue or tiredness was recognised by all participants as a secondary consequence of their motor difficulties and many were aware that being tired also made them more susceptible to tripping or falling, for example:

I get so tired and as a consequence this increases my chances of stumbling - so it is a vicious circle.

### Activity Limitations: Difficulties Executing Daily Tasks

As suggested by the DSM-5 [1] diagnostic criteria for DCD, all participants faced challenges with daily functioning. Undertaking personal care activities was seen to potentially increase their chances of becoming unbalanced. These activities were either avoided, as in the case of bathing, or adapted to ensure their safety. With reference to bathing:

The combination of having to stand on one leg to get in and out and a wet surface is something I avoid.

I don’t use the bath anymore, I tend to shower to ensure safety getting in and out.

And dressing:

I have to sit to dress so I don’t fall over.

Dressing also highlighted an ongoing reliance on vision to guide movements, which was also noted when discussing moving around at night and carrying large objects that obscured the view of their feet:

I hate pulling things over my head, the tugging makes me wobbly and I don’t cope well if my sight is obscured.

I throw the washing down the stairs for example, rather than carry it as it blocks my view of my feet.

Activities that required stretching which moved them beyond their base of support were also highlighted as potentially ‘risky’.

Hanging the washing on the line, as reaching up puts me off balance.

Concerns were expressed about tasks undertaken away from home with one participant describing her concerns about the walk to work:

I am aware that I become tense as I begin the descent down from the car park towards the building, it is particularly bad in the autumn and winter when it becomes slippery.

All expressed frustration with not feeling competent to do some tasks or at speed:

Things can just take so long to complete because I know I need to slow down to do things to remain upright.

Many felt they were slowed down by their inability to do two things at once, with one participant exclaiming her amazement at others’ ability to dual task:

I look at people walking and texting in wonder as I know I would definitely fall or walk into something.

### Participation Restrictions: Difficulties in Wider Social Situations

Limitations at an activity level did appear to alter their ability to participate, although these were not always overtly discussed. Many discussed participation in terms of how they had made conscious decisions to ensure they could complete roles such as being an employee. For example, the way they coped in the work place because of their mobility difficulties and consequential fatigue.

I aim always to arrive earlier than my colleagues so I can park nearer the building to avoid the steps and slope down from the top car park.

As soon as I could afford to, I dropped a day just to help manage the fatigue. Before that I was spending all weekend trying to recharge my batteries and missed out on family time.

Interestingly, whilst DCD is often referred to as a hidden disability, they discussed their concerns about how other colleagues may view them, with one expressing:

I am sure people think I am a hypochondriac when I tell them I have sprained my ankle again or when I complain about being tired.

Adding to this, they suggested that others might see them as antisocial as they are often unable to participate in the social aspects of work, which they felt helped to consolidate work relationships*.* Almost all participants discussed spending leisure time with family members ‘*who know what I am like*’.

Some did explain that their fear of falling stopped them completing certain past times:

I am fearful of going out on my own with him (the dog), incase I fall.

And another explained that although they try to carry on with leisure activities, their mobility difficulties did impact on their enjoyment:

I really like hill walking, however I have to concentrate so hard on where I am placing my feet and not falling over I end up not enjoying the walk at all.

Participation in more active leisure pursuits with others outside of the family was also carefully selected*:*

I look at friends skiing and ice skating and think that looks fun but could never think of joining in.

I tend to choose to do physical activities on my own as I know I can’t compete in team activities.

When considering their participation in their roles as partner and parent, a number described incidents when they had been concerned about their and others’ safety with one participant explaining how her difficulties with mobility made her continue to worry about her and her child’s safety:

I had to have an additional hand rail put up on the stairs as I became so anxious about falling when carrying the little one down stairs.

Two other participants discussed how they and their partners divided the household chores to avoid trip and falls hazards, which at times caused real frustration:

Whilst we make it work…. I do get a little upset at times when I think, surely, I should be able to do basic things like hanging the washing on the line without thinking I am going to fall.

### Personal Factors

Whilst reviewing and coding the scripts, it became obvious that a fourth theme needed to be considered—personal factors. These were the personal attributes that these participants drew upon to mediate the disabling impact of DCD on daily life. Examples of these were the planned adaptations that were referred to throughout the interviews and help to illustrate how they positively utilised other skills to manage the effects of their motor difficulties. For example, a number of participants discussed how they had problem-solved an issue and had rearranged their home to alleviate the risk of trips and falls:

I have rearranged my kitchen cupboards, so I don’t over stretch when getting things out…when I have my new kitchen I want an eye level oven so I don’t have to call my partner to lift things up from the oven.

Re-arrangements extended to their wardrobe and the modification of footwear was commonly discussed:

I tend to pick flatties for work if I know I may have to move quickly and have bought wedges, that have a bigger base to stand on when going out posh.

Talking about high heels one stated:

For years I kept the ones I had because they were lovely but knew I could never walk in them safely.

The need to carefully plan activities was seen as a necessity to manage the effects of adverse environments on their safety:

I am not good in the dark or when somewhere is badly lit so in the cinema I just book an aisle seat near the door in case I need to go out to the loo.

When they could not plan, they identified how they drew on past experience to help to make quick management decisions if adverse situations arose:

I will wait for a later bus if is full and it would mean I have to stand up.

Avoidance and delegation was something that was mentioned as an adaptation, although this was often discussed as a being a negative but necessary strategy, as reliance on others was something all had tried to avoid.

It was noticeable that often during the interviews, a humorous tone was adopted to discuss their motor difficulties and its impact on their lives:

I am sure the door frames move in my house as I regularly bump into them and the corner of the table.

## Discussion

The purpose of this study was to explore the negative or disabling outcomes of living with significant motor difficulties on the lives of adults with a DCD diagnosis. The primary aim was to explore how adult participants viewed their motor impairments and the potential consequential activity limitations and participation restrictions. This was done to try to gain a fuller understanding of the lived experience of having functional mobility difficulties.

The findings from this study would appear to offer further evidence that for some individuals with DCD, the effects of having significant motor difficulties negatively impact on daily life, well into adulthood. The participants in this study were aware of and lived with the negative impact on their functional mobility of having motor impairments, clearly perceiving, based on past experience, that they were at times at risk of tripping or falling. They were able to describe and had attempted to explore for themselves why they had difficulties with motor proficiency offering similar explanations to those identified by Cousins and Smyth [5] and Wilmut et al. [11•]. In addition, they were able to expand upon these explanations and offer a description of how this impacted on the decisions they made to engage in certain activities based on whether they felt safe to try or not.

The participants’ demographic information suggested that all the participants were undertaking activities that enable them to participate in a range of typical adult roles, such as being an employee, partner and for some a parent, in mainstream work and leisure environments. The interview findings reiterated that they do take part in the full range of activities that are necessary to fulfil their roles at home and in work. There was evidence throughout, however, that they made conscious decisions about how to approach these activities to minimise the impact of their motor impairments on their ability to successfully and safely undertake them. This included managing both the physical, temporal and social environment. As adults, unlike adolescence and children, they seemed to have some ability to ‘create more positive situations for themselves’ [16]. However, the management of impaired mobility through adaptation was seen not to be without its frustrations and the effort employed was often to the determent of their energy and anxiety levels, which for this group were body function impairments that could at times be as disabling as their primary motor impairments [17].

Whilst there may be less available choice with respect to the activities adults need to undertake in relation to work, as a group, the participants would appear to make considered decisions about what they undertake in the home and during their leisure time. They were noted to choose to avoid some group leisure activities in favour of individual ones or to delegate household chores to others. The choices made would appear to ensure a better match between their motor abilities and activity success, which reiterates the findings of Missiuna et al.’s [16] study of adolescents with DCD who were noted to search out opportunities to participate in an environment that demands less motor proficiency. However, with respect to leisure participation, this avoidance appeared not only to be related to their self-awareness of their level of motor proficiency and any associated fear of tripping and/or falling but also to be based on concerns about how they may be perceived by others. This anxiety related to how others perceive them is also reflected as a concern in Fitzpatrick and Watkinson [18] study of children with DCD, which highlighted that avoidance may be considered a self-protective strategy. Interestingly, in this study, the participants’ reluctance to take part in physically demanding activities did not extend to those undertaken with family members, which suggests that the social environment can mediate the gap between their movement abilities and the pleasure and success of undertaking certain activities. Family members were perceived to understand and accept the individual’s motor difficulties and as such offer a safe social environment to try out or continue to preserve with more challenging physical activities.

Whilst all the individuals were experiencing difficulties with functional mobility and its impact on daily life, all were actively engaged in a wide range of age- and ability-appropriate work, home and leisure activities. The findings from this study would suggest that a range of personal factors mitigated the impact of poor motor proficiency. All participants in their descriptions demonstrated that they consistently employed problem-solving skills to make adaptations to the environment to afford them the opportunity to achieve success in an activity. Being able to self-evaluate their own skill level as well as make an analysis of the demands of an activity evidently meant that, on the whole, they were making accurate decisions about whether they could safely and successfully undertake certain pursuits. Other factors such as the use of protective personal attributes, identified in resilience literature, for example Luthar [19], act as important mediators when facing adversity and were adopted by participants to help mediate the effects of having poor motor proficiency. These protective personal factors included the use of humour when describing their own attributes as well as a general acceptance that they would need higher levels of persistence and determination when compared to their peers to achieve success in activities that require good motor control.

## Conclusion

This study was able to elicit how adults with DCD live and manage their motor difficulties when they impact negatively on their functional mobility. Positively, the participants in this study were found to be involved in a wide range of age- and ability-appropriate activities and levels of participation. They were aware of their motor impairments and the impact they had on functional mobility and through discussion were able to identify how they made regular adaptations to the physical, temporal and social environment to ensure they successfully manage the activities they are required to fulfil in their various adult roles. These adaptations draw on and utilise personal attributes such as problem-solving skills to plan and moderate the effects of poor motor proficiency. Additionally, adults with DCD appear to be constantly re-evaluating their own level of motor skills with respect to the activity requirements to ensure that they limit their risk of tripping and falling. The use of humour and high levels of perseverance and determination were also evident as protective factors that mediated the daily challenges they face. However, throughout was evidence that whilst they were, on the whole, successfully managing their functional mobility, participants described the additional effort and time needed to manage their disability and this often resulted in increased levels of fatigue and heightened levels of anxiety that were considered to be the secondary and hidden consequences of living with DCD.

The hidden consequences of living with difficulties with functional mobility go beyond the constant need for vigilance when managing everyday activities. There is therefore a need to measure levels of anxiety and fatigue as a part of any functional assessment. In addition, to ensure that we equip children and young people to manage the ongoing effects of living with motor difficulties, it would be beneficial to foster the personal attributes and protective factors that help adults to manage active engagement in the necessary activities needed for adult life.

Whilst it was never the intention of this qualitative study, no definitive conclusions can be drawn from the findings due to the small sample size. The recruitment procedures undertaken did lead to the sample being made up of mainly females, with all participants being well educated to further or higher education level, which could help to explain why they were able to positively manage the difficulties encountered due to poor functional mobility. The participants involved had sought or were referred for a clinical diagnostic assessment within the previous 5 years of the study commencing. Whilst none of the participants had received ongoing clinical intervention and the screening process eliminated ADHD and those who were on medication that could impact on functional mobility, the participants may have had co-occurring characteristics of other neurodevelopmental or psychological disorders that could impact on functional mobility.

## Footnotes

1 Functional mobility describes a person’s ability to move around in his or her environment.
